# NCF1/2/4 Are Prognostic Biomarkers Related to the Immune Infiltration of Kidney Renal Clear Cell Carcinoma

**DOI:** 10.1155/2021/5954036

**Published:** 2021-10-18

**Authors:** Yifei Chen, Fei He, Ruhua Wang, Menglin Yao, Yarui Li, Dan Guo, Shuixiang He

**Affiliations:** ^1^Department of Gastroenterology, First Affiliated Hospital of Xi'an Jiaotong University, Shaanxi 710061, China; ^2^Department of Urology, The First Affiliated Hospital of Xi'an Jiaotong University, Xi'an, Shaanxi 710061, China

## Abstract

Neutrophil cytoplasmic factor 1/2/4 (NCF1/2/4) belongs to the NADPH oxidase complex, which is a cytoplasmic component, and its polymorphism is the main factor related to autoimmune diseases, which is probably caused by the regulation of peroxide. They also play a role in tumor growth and metastasis. This research is aimed at evaluating the biological function and prognostic role of NCF1, NCF2, and NCF4 genes in kidney renal clear cell carcinoma (KIRC) by using multiple online bioinformatics website, including Oncomine, GEPIA, UALCAN, Kaplan–Meier Plotter, TIMER, TISIDB, cBioPortal, LinkedOmics, GeneMANIA, and DAVID databases. The mRNA levels of NCFs were higher in KIRC tissues than in normal tissues. The overexpression of NCFs was significantly correlated with advanced pathological grades and individual cancer stages in KIRC. Meanwhile, the expressions of NCFs played an important role in the tumorigenesis and progression of KIRC. Prognostic value analysis suggested that high transcription levels of NCF1/4 were associated with poor overall survival in KIRC patients. In addition, results from the LinkedOmics database showed that the KEGG pathway related to NCFs mainly focused on immune activation and immune regulation function. NCF genetic alterations, including copy number amplification, missense mutation, and deep deletion, could be found through the cBioPortal database. Further, NCF expression was significantly correlated with infiltration levels of various immune cells as well as immune signatures. Protein-protein interaction network and enrichment analysis of NCF1/2/4 in KIRC showed that NCF coexpressed genes mainly associated with diverse immune marker sets showed significance. Overall, these results indicated that NCFs could be prognostic biomarkers as well as effective targets for diagnosis in KIRC.

## 1. Introduction

In recent years, the incidence of malignant kidney tumors has been increasing. In 2019, approximately 73,820 Americans were diagnosed with kidney cancer [1], and nearly 15,000 died of the disease [2, 3]. It is the seventh most common cancer among men and the ninth most common cancer among women [4]. Kidney cancer represents several different types of cancer, which have different histology, clinical course, and response to treatment [5]. Clear cell carcinoma is the most common histological type of kidney cancer (75%) [6] and one of the most aggressive types [7]. Because the kidney is located deep in the body, its clinical symptoms usually appear in the late stage, so the 3-year survival rate in the kidney renal clear cell carcinoma (KIRC) in the population is less than 5% [8]. Until recently, we still lacked effective systemic therapies for KIRC, and surgery was the main treatment method [9]. In addition, the prognosis of KIRC is not good. One-third of patients will have local or distant metastases, and about one-quarter of patients undergoing radical surgery have recurred tumors at a distance [10]. Due to the high morbidity, high mortality, and difficulty of early diagnosis of KIRC, it is important to evaluate carcinogenic mechanisms and explore potential drug targets and molecular markers that have a prognostic value that affects the immune response of KIRC patients.

Neutrophil cytoplasmic factor 1 (NCF1), neutrophil cytoplasmic factor 2 (NCF2), and neutrophil cytoplasmic factor 4 (NCF4) are also referred to as p47phox, p67phox, and p40phox, respectively. They belong to the NADPH oxidase complex, which is a cytoplasmic component, and its polymorphism is the main factor related to autoimmune diseases, which is probably caused by the regulation of peroxide [11]. In the case of inflammatory stimulation, NADPH oxidase is activated; phosphorylation of NCF1 (p47phox) leads to the assembly of NCF2 (p67phox) and NCF4 (p40phox) into an active oxidase complex p22phox/gp91phox. In this process, it promotes the conversion of oxygen (O_2_) into superoxide ions (O_2_^−^) and hydrogen peroxide (H_2_O_2_), which are all reactive oxygen species (ROS) [12]. Studies have confirmed the correlation of these genes with chronic granulomatous disease [13], Crohn's disease [14], and autoimmune arthritis [15]. They also play a role in tumor growth and metastasis [16], such as Hodgkin's lymphoma [17]. However, their relationship with tumors in many other fields has not been explored, so this article is aimed at evaluating the biological function and prognostic role of NCF1, NCF2, and NCF4 genes in KIRC.

## 2. Materials and Methods

### 2.1. Oncomine Database Analysis

The mRNA levels of NCFs in various cancers were identified in the Oncomine database. The Oncomine database (http://www.oncomine.org) is an online cancer microarray database and synthetic gene-wide data-mining platform [18]. We compared the transcriptional levels of NCFs in different cancer tissues with their corresponding adjacent normal controls from the Oncomine database, using Student's *t*-test to generate a *p* value. Cutoffs of *p* value and fold change were defined as 0.05 and 2, respectively.

### 2.2. Gene Expression Profiling Interactive Analysis

Gene Expression Profiling Interactive Analysis (GEPIA) (http://gepia.cancer-pku.cn/) integrates a tremendous amount of tumor and nontumor samples from The Cancer Genome Atlas (TCGA) and the Genotype-Tissue Expression (GTEx) database, providing differential expression analysis, correlation analysis, and patient survival analysis online [19]. In our study, GEPIA was used to analyze the expression of NCFs in kidney renal clear cell carcinoma with corresponding breast tissues. The cutoff of *p* value was 0.05 and log_2_FC was 1 (fold change was 2). We also obtained the top 100 similar expression protein-coding genes with certain NCFs in KIRC Tumor dataset by similar gene module for Gene Ontology (GO) and Kyoto Encyclopedia of Genes and Genomes (KEGG) analysis.

### 2.3. UALCAN Database Analysis

UALCAN (http://ualcan.path.uab.edu) is a comprehensive web resource based on TCGA database level 3 RNA-seq and clinical data from 31 cancer types [20]. It can be used to estimate the relative transcriptional expression of query genes between tumor and normal samples as well as relative clinicopathologic parameters on patient survival. In this study, UALCAN was used to analyze the association between mRNA expressions of NCFs grouped by known prognostic factors (individual cancer stages, tumor grade, and KIRC subtype) in kidney renal clear cell carcinoma.

### 2.4. Kaplan–Meier Plotter Database

The research on the NCF prognostic value was performed in the KM-plotter database, which could evaluate the survival of more than 50,000 genes in 21 cancer types [21]. The ccRCC dataset with 530 samples was selected to explore the expression profile of NCFs on ccRCC overall survival (OS). Furthermore, the hazard ratio (HR), log-rank *p* value, and survival plots were computed and output by the website automatically.

### 2.5. LinkedOmics Database Analysis

LinkedOmics is a publicly available portal that included multiomics data from 32 TCGA cancer types [22]. The “Linkfinder” module was used to perform the volcano map showing the NCF association results and heat map showing the coexpressed genes of NCFs. The “LinkInterpreter” module was used to perform GO and KEGG analysis of NCFs based on gene set enrichment analysis (GSEA). The criterion about GSEA is as follows: the minimum number of genes (size) is 3 and a simulation is 500. The top 5 terms of GO and KEGG analysis were exhibited. Gene terms with *p* value < 0.05 and false discovery rate (FDR) < 0.05 were considered significant.

### 2.6. cBioPortal Data Analysis

The cBioPortal for Cancer Genomics is a comprehensive web resource that can visualize and analyze multidimensional cancer genomic data [23]. Copy number variation (CNV), mutations, and the clinic outcomes of the gene types in KIRC were evaluated according to the online tools of cBioPortal. The *p* value set as 0.05 was considered significantly different.

### 2.7. TIMER Database Analysis

The TIMER database is a comprehensive resource for systematical analysis of immune infiltrates across diverse cancer types [24]. Associations between NCF expression and TIIC infiltration levels were analyzed via TIMER, a website tool for analysis of gene-specific correlation with TIICs. TIICs included B cells, CD4^+^ T cell, CD8^+^ T cell, macrophages, neutrophils, and dendritic cells.

### 2.8. TISIDB Database Analysis

Through the TISIDB online platform (http://cis.hku.hk/TISIDB/), the expression levels of NCFs in the six immune subtypes were analyzed [25].

### 2.9. Protein-Protein Interaction Network Construction

The STRING (http://string-db.org, version 11.0) database was used to predict the PPI network of DEGs and analyze the interactions between proteins [26]. GeneMANIA is an interactive and visual online protein-protein interaction (PPI) prediction tool, which provides the customizable function of the detection of genes with similar functions. GeneMANIA is a prediction website tool for analyzing genetic and protein interactions, coexpression, pathways, colocalization, and domain-protein similarity of target genes [27]. In this study, we analyzed the relationship between NCFs and their interactive genes by the GeneMANIA database and the STRING database.

### 2.10. DAVID Database

DAVID contains a comprehensive set of functional annotation tools for better clarifying the biological functions of target genes [28]. In this work, Gene Ontology (GO) and Kyoto Encyclopedia of Genes and Genomes (KEGG) pathway enrichment analyses of NCFs and their coexpressed genes (100) were conducted using the DAVID tool. The cutoff value for significant GO terms and KEGG pathways was a false discover rate (FDR) of <0.05.

### 2.11. Cell Culture and Transfection

We purchased human normal renal tubular epithelial cell line HK-2 and kidney cancer cell line 786-O from the Cell Bank of Type Culture Collection of Shanghai Institute of Cell Biology. The cells were cultivated aseptically within DMEM medium (Gibco, San Diego, CA) that contained 10% fetal bovine serum (FBS) and maintained at 37°C in a humidified incubator with 5% CO_2_. For H_2_O_2_-induced oxidative stress, 500 *μ*M H_2_O_2_ was added to the culture medium. Small interfering RNA of targeted NCF4 RNA (si-NCF4, 50 nM) were designed and synthesized by RiboBio (China). Cell transfection was performed with the usage of Lipofectamine 2000 (Invitrogen), according to the protocol provided by the supplier.

### 2.12. Determination of ROS Formation

ROS formation was measured using H_2_DCFDA (Invitrogen), after pretreatment with 4 *μ*mol H_2_DCFDA in serum-free and pH indicator-free medium for 30 min. Then, incubation cells were washed twice with PBS and analyzed (FACSCalibur, Becton Dickinson). Relative ROS level was determined by fluorescence intensity.

### 2.13. Cell Counting Kit-8 (CCK-8) and Colony Formation

The Cell Counting Kit-8 (Beyotime Biotechnology, Jiangsu, China) was used to measure cell proliferation according to the manufacturer's protocol. Cells were seeded into 96-well plates at a density of 1 × 10^3^ cells per well and determined every 24 h. The way to determine was by adding 10 *μ*l of CCK-8 solution into each well and then culturing for 4 hours in an incubator. Four hours later, the absorbance value (*A*) of cells was detected at the wavelength of 490 nm. For colony formation assays, cells were seeded into six-well plates at the density of 500/well and were cultivated continuously at 37°C for 14 d. Then, the colonies were washed and stained with 0.2% crystal violet solution for 2 hours.

### 2.14. Wound Healing and Transwell Assays

When the cell density attained 90% after 24 hours of transfection in 6-well plates, the wound was scratched by a 10 *μ*l sterile tip and then washed off the floated cells. Under the microscope, photographs of the wound were taken at 0 and 36 hours. For transwell migration assays, 1 × 10^5^ cells were plated into the upper chambers of a transwell apparatus (24-well insert, 8 *μ*m pore size, Corning) with 200 *μ*l of serum-free medium, and the bottom chamber was filled with 700 *μ*l medium supplemented with 10% FBS. After 24 h of incubation, the cells on the lower surface of the membrane were stained, photographed, and counted in six random fields per group using a microscope.

### 2.15. RNA Extraction and Western Blotting

According to the manufacturer's instructions, the total RNA was extracted from cultured cells by using Trizol reagent (Invitrogen, USA). Then, the cDNAs were synthesized following the protocol of PrimeScript™ RT Master Mix (Takara). Quantitative real-time PCR was conducted using SYBR Premix Ex Taq™ II (Takara) on Thermal Cycler CFX6 System (Bio-Rad). The relative transcriptional levels of target genes were calculated by using the 2−*ΔΔ*Ct method. The RT-qPCR primer sequences were as follows: NCF1 forward primer 5′-ACGAGAGTGGTTGGTGGTTC-3′ and reverse primer 5′-TGTAGGCTTTGATGGTGACG-3′, NCF2 forward primer 5′-GCGCTAGGCTGGGACCTTGAAGCC-3′ and reverse primer 5′-GTCTTGAAGAAGGGCAGTGATAAC-3′, and NCF4 forward primer 5′-TGAACAGCTTCCGGATGATG-3′ and reverse primer 5′-TGAAGCCTCTCTTCTCCTCGAT-3′. Western blot was performed by adding lysis buffer into cells and total protein was extracted. Equal amounts of protein samples were added to SDS/PAGE for conducting electrophoresis. Next, after being blocked for 1 hour with 5% defatted milk, the membranes with proteins were incubated in a primary antibody against NCF4 (Invitrogen), E-cadherin, *α*-catenin, vimentin, MMP9, and *β*-actin (Cell Signaling Technology), overnight at 4°C. Then, the membranes were incubated with the secondary antibodies and the proteins in the membranes were visualized by ECL after washing with TBST. *β*-Actin was used as a reference.

### 2.16. Statistical Analysis

Statistical analyses were performed using the SPSS 23.0 statistical software (IBM, USA), GraphPad Prism software (7.0). The quantitative data were shown as mean ± standard deviation (SD) and were compared through Student's *t*-test. The enumeration data enlisted as percentages were compared via the chi-square test. It would be considered as statistical significance when *p* < 0.05.

## 3. Result

### 3.1. Aberrant Expression of NCF1, NCF2, and NCF4 in Multiple Cancer Types and In-Depth Verification in KIRC

**Figure 1:**
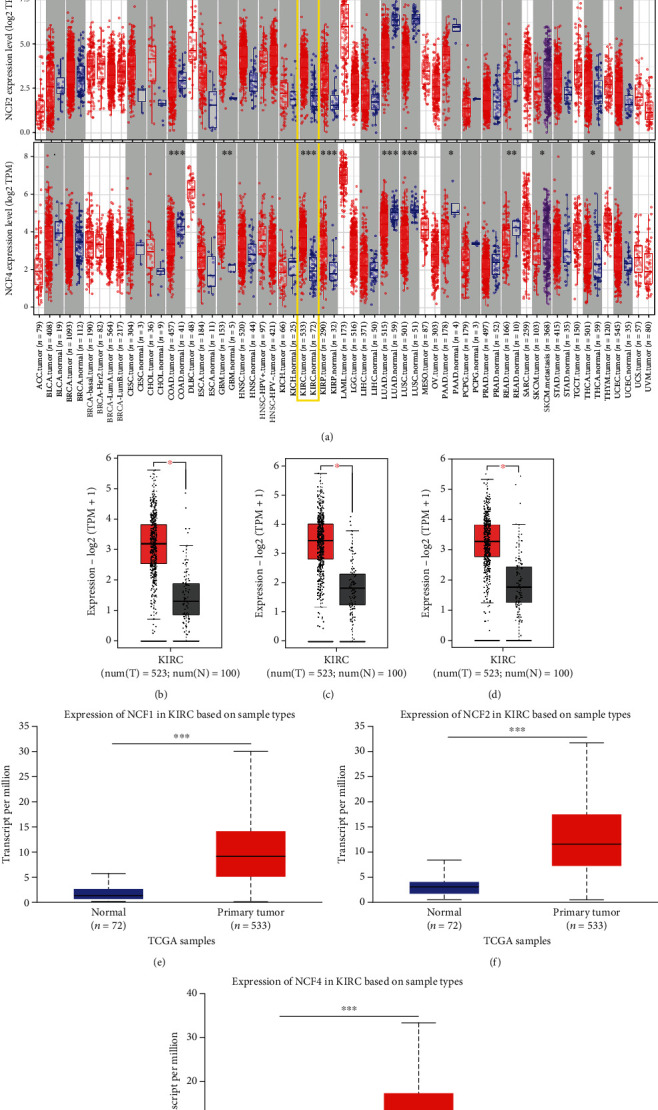
The expression levels of NCF1, NCF2, and NCF4 in KIRC were higher than those in the respective adjacent normal tissues. (a) Expression levels of NCF1, NCF2, and NCF4 in different types of tumors from TCGA database were determined using TIMER 2.0. (b–d) Expression levels of NCF1, NCF2, and NCF4 in KIRC tissues compared with normal tissues using the GEPIA database. (e, f) Expression levels of NCF1, NCF2, and NCF4 were expressed as box plots in normal individuals and patients with KIRC using the UALCAN database. ^∗^*p* < 0.05, ^∗∗^*p* < 0.01, and ^∗∗∗^*p* < 0.001.

In order to preliminarily evaluate the role in tumorigenesis, we analyzed the different expression levels for NCF1, NCF2, and NCF4 between tumor and adjacent normal tissues in all TCGA tumors (Figure 1(a)). Our results showed that the levels of NCF expression are significantly downregulated in multiple cancer types including LUAD (lung adenocarcinoma) and LUSC (lung squamous cell carcinoma), but were higher in GBM (glioblastoma multiforme), KIRC (kidney renal clear cell carcinoma), and KIRP (kidney renal papillary cell carcinoma). These data show that NCF1, NCF2, and NCF4 have different expression levels in different cancers, suggesting that NCFs exerted diverse functions in various cancers. Differential expression was observed between tumor and normal tissues for NCF1, NCF2, and NCF4 in KIRC data from TCGA. The results indicated that NCF1, NCF2, and NCF4 were upregulated in KIRC compared with adjacent normal tissues (*p* < 0.001; Figures 1(b)–1(d)). Analysis of TCGA-KIRC samples in the UALCAN database revealed that mRNA expression levels of NCF1, NCF2, and NCF4 were significantly higher in KIRC tissues than in control tissues (*p* < 0.001; Figures 1(e)–1(g)).

### 3.2. The Levels of NCF1, NCF2, and NCF4 Expression in Subgroups of Patients with KIRC

**Figure 2:**
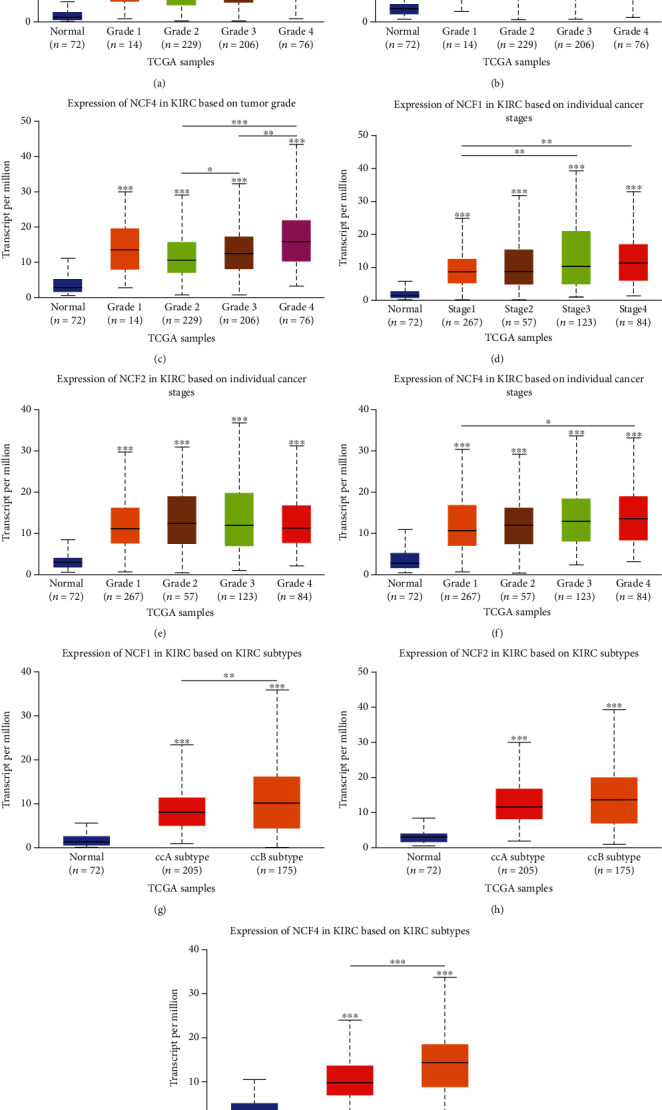
The expression levels of NCF1, NCF2, and NCF4 in subgroups of patients with KIRC. Relative expression of NCF1, NCF2, and NCF4 in (a–c) normal individuals and patients with KIRC at different grades, (d–f) normal individuals and patients with KIRC at different stages, and (g–i) normal individuals and patients with KIRC at different subtypes. ^∗^*p* < 0.05, ^∗∗^*p* < 0.01, and ^∗∗∗^*p* < 0.001.

To study various clinicopathological characteristics, we further analyzed TCGA-KIRC samples in the UALCAN database. We next explored the different expression levels of the 3 genes in KIRC, stratified according to the cancer stage and pathological grade. The results demonstrated that the expression of the 3 genes was higher in KIRC tissues than in normal tissues based on different pathological grades and individual cancer stages. Therefore, expression levels of NCF1, NCF2, and NCF4 may serve as potential diagnostic markers in patients with KIRC (Figures 2(a)–2(f)). In addition, we also analyzed the mRNA expression levels of the NCFs in different subgroups of primary KIRC patients and healthy people. NCF1, NCF2, and NCF4 are significantly higher in ccA and ccB subtypes than normal tissues, and the expression of NCF1 and NCF4 in ccB subtypes is significantly higher than that in ccA subtypes (Figures 2(g)–2(i)). Therefore, the results indicated that the expression of these genes plays an important role in the tumorigenesis and progression of HCC.

### 3.3. Clinicopathological Characteristics and Prognostic Significance of NCF1, NCF2, and NCF4 Expression in KIRC

**Figure 3:**
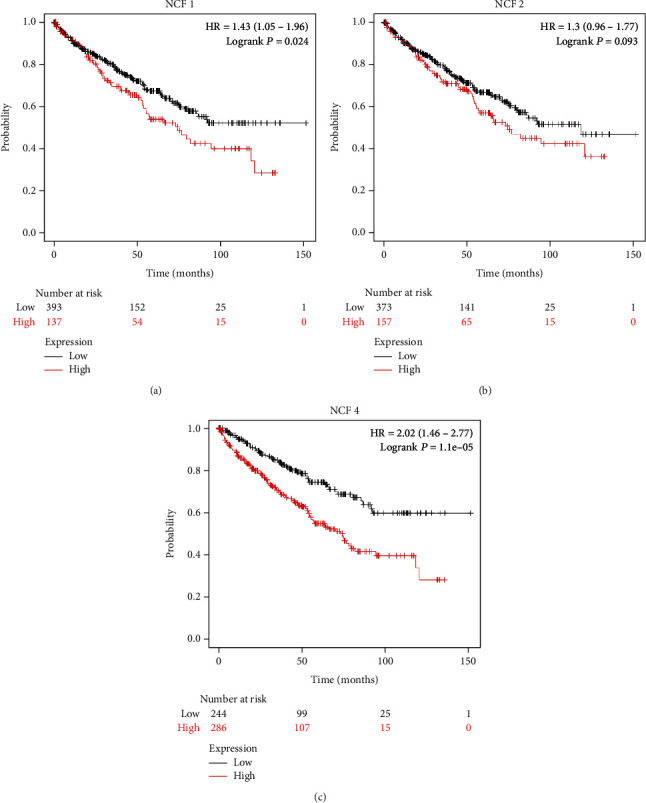
NCF1, NCF2, and NCF4 are associated with the overall survival (OS) rate in KIRC. (a–c) Kaplan–Meier survival curves comparing high- and low-expression levels of NCF1, NCF2, and NCF4 in KIRC using Kaplan–Meier Plotter database. Survival curves based on overall survival (OS).

The transcriptome RNA-seq data from TCGA databases for 530 KIRC patients was collected. The clinicopathological characteristics of these kidney cancer patients are depicted in Table 1. Next, we sought to investigate the prognostic significance of NCF1, NCF2, and NCF4 expression in KIRC. The association between NCF1, NCF2, and NCF4 expression levels and the survival outcomes of KIRC patients was assessed using Kaplan–Meier survival curves. The results showed that patients were divided into two groups based on the expression values of NCF1, NCF2, and NCF4 in each cohort (autoselect the best cutoff). The group with high NCF1 and NCF4 expression had significantly shorter overall survival (OS) compared to the group with low expression of NCF1, NCF2, and NCF4 based on TCGA data using the GEPIA database (Figure 3). These results strongly highlight the prognostic value of NCF1, NCF2, and NCF4 in KIRC.

### 3.4. Alterations of NCF1, NCF2, and NCF4 Expression Networks in KIRC

**Figure 4:**
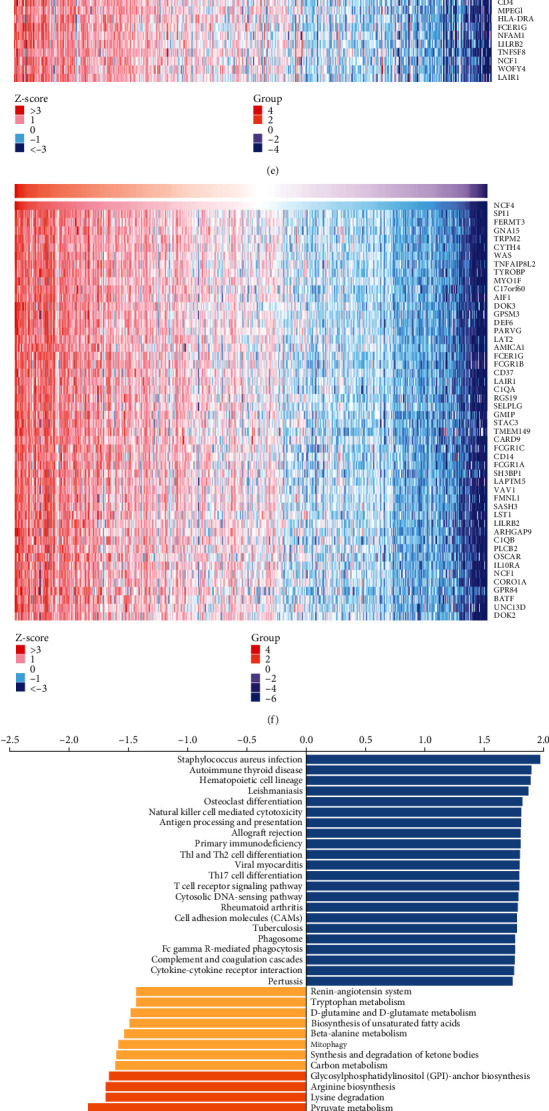
Differentially expressed genes and gene set enrichment analysis (GSEA) with NCF1, NCF2, and NCF4 high and low expression groups. (a–c) The NCF1, NCF2, and NCF4 highly correlated genes identified by Pearson test in KIRC cohort. (d–f) Heat map showing positive and negative correlations of the first 50 genes with NCF1, NCF2, and NCF4 in KIRC. Red indicates positively related genes, and blue indicates negatively related genes. (g–i) Significantly enriched KEGG pathways of NCF1, NCF2, and NCF4 in KIRC.

Since gene regulation networks reflect common genetic risk factors that compose functional relationships, we investigated the regulatory factors related to NCF1, NCF2, and NCF4 in KIRC. Figures 4(a)–4(c) show genes highly coexpressed with NCF1, NCF2, and NCF4 based on Pearson correlation; genes positively and negatively correlated with NCF1, NCF2, and NCF4 are marked in the dark red and dark blue dots, respectively (FDR < 0.01). The top 50 genes showing significant positive and negative correlation with NCF1, NCF2, and NCF4 are shown in heat maps (Figures 4(d)–4(f)).

In addition, results from the LinkedOmics database showed that the KEGG pathway related to NCFs mainly focused on immune activation and immune regulation function (Figures 4(g)–4(i)).

High expression of NCF1 was associated with adaptive immune response (BP category), immunological synapse (CC category), MHC protein binding (MF category), and *Staphylococcus aureus* infection (KEGG category). High expression of NCF2 was associated with detection of biotic stimulus (BP category), MHC protein complex (CC category), pattern recognition receptor activity (MF category), and *Staphylococcus aureus* infection (KEGG category). High expression of NCF4 was associated with adaptive immune response (BP category), immunological synapse (CC category), immunoglobulin binding (MF category), and *Staphylococcus aureus* infection (KEGG category). More enrichment pathways are shown in Table 2.

### 3.5. Genomic Alterations of NCF1, NCF2, and NCF4 in KIRC

**Figure 5:**
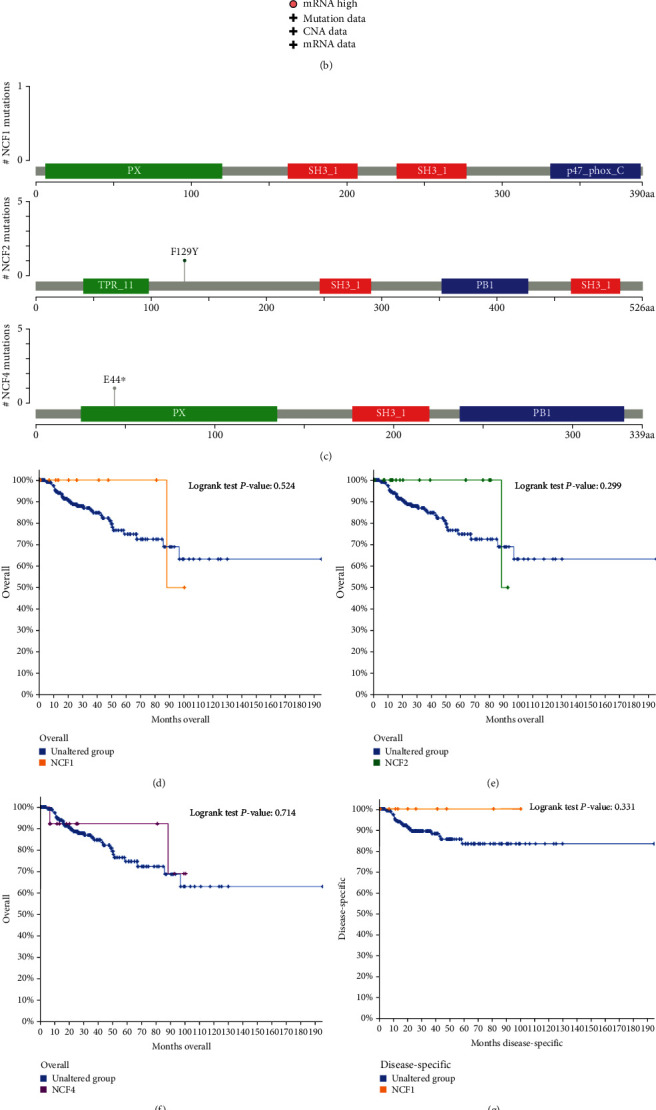
Genomic alterations of NCF1, NCF2, and NCF4 in KIRC. (a) OncoPrint of NCF1, NCF2, and NCF4 alterations in KIRC. Different types of genetic alterations highlighted in different colors, including mutation, mRNA high, and deep deletion. (b) Mutation frequency and copy number variations in the TCGA-BRCA dataset were determined using the cBioPortal website. (c) The mutation site legend of NCF1/2/4. (d–i) Association between cases with NCF1, NCF2, and NCF4 CNA status and overall survival (OS) and disease-specific survival (DSS) of patients with KIRC.

The frequency and types of genetic alterations in NCF1, NCF2, and NCF4 in patients with HCC were analyzed by using the cBioPortal database. A total of 274 KIRC cases from TCGA were explored. NCF1, NCF2, and NCF4 were altered in 4%, 7%, and 5% of KIRC cases, respectively (Figure 5(a)). NCF1 mutation consisted of 4.01% mRNA high (11 cases). NCF2 mutation consisted of 0.36% mutation (1 case), 0.36% deep deletion (1 case), and 5.84% mRNA high (16 cases). NCF4 mutation consisted of 0.36% mutation (1 case), 0.36% deep deletion (1 case), and 4.01% mRNA high (11 cases) (Figure 5(b)). NCF2 and NCF4 have existed mutations in the protein functional domain (Figure 5(c)). However, the copy number alteration status of NCF1, NCF2, and NCF4 was not significantly associated with the overall survival (OS) and disease-specific survival (DSS) of KIRC (Figures 5(d)–5(i)). In KIRC patients, the probability of mutations in NCF1, NCF2, and NCF4 is not high. We did not find that mutations may have beneficial or harmful effects on survival in this small sample of data. Therefore, genomic alterations of these genes could not be considered as poor prognosis factors in KIRC patients.

### 3.6. Correlation between NCF Expression and Immune Infiltrating Level in KIRC

**Figure 6:**
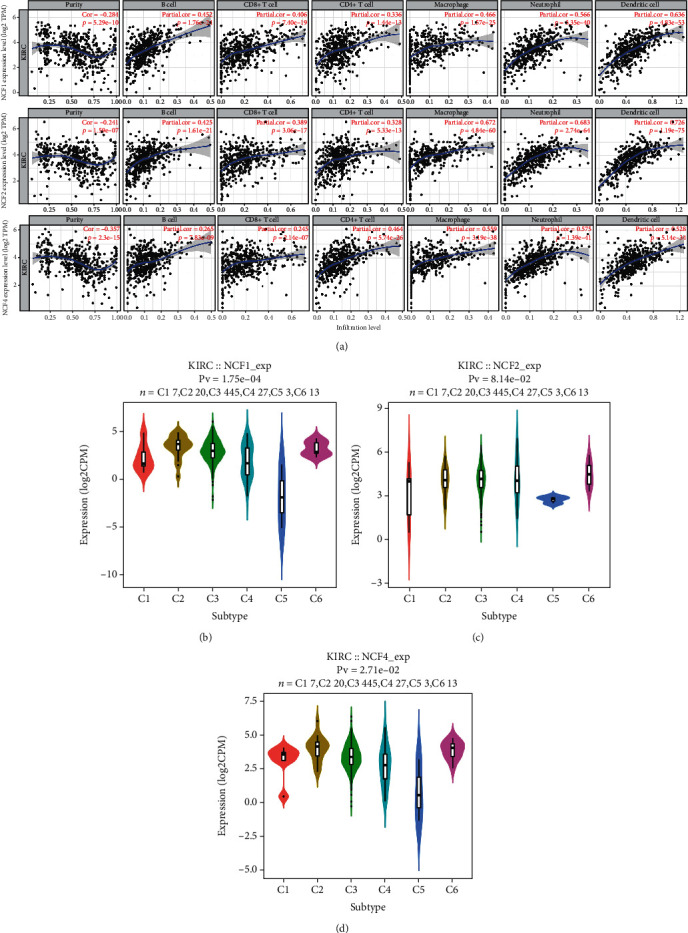
Correlation between NCF expression and immune infiltrating level in KIRC. (a) NCF1, NCF2, and NCF4 expression levels were significantly related to tumor purity and significant positive correlations existed with immune infiltration cells including CD4^+^ T cells, CD8^+^ T cells, B cells, neutrophils, macrophages, and DCs in KIRC. (b–d) Correlation of NCF1, NCF2, and NCF4 expression and immune subtypes (C1-C6: wound healing, IFN-gamma dominant, inflammatory, lymphocyte depleted, and TGF-*β* dominant) in KIRC.

The survival of patients in several cancers is determined by the number and activity of tumor-infiltrating lymphocytes. Therefore, the TIMER database was used to investigate the relationship between the levels of immune infiltrating and the expressions of NCF1, NCF2, and NCF4 in KIRC patients. As shown in Figure 6(a), high levels of NCF1, NCF2, and NCF4 mRNA expression had a significantly negative correlation with tumor purity (NCF1, *r* = −0.284, *p* < 0.001; NCF2, *r* = −0.241, *p* < 0.001; and NCF4, *r* = −0.357, *p* < 0.001) in KIRC.

Overexpression of each of these genes was significantly associated with higher immune cell infiltration levels. Specifically, the NCF1, NCF2, and NCF4 expression level was positively correlated with infiltration levels of CD8^+^ T cells (NCF1, *r* = 0.406, *p* < 0.001; NCF2, *r* = 0.389, *p* < 0.001; and NCF4, *r* = 0.245, *p* < 0.001), CD4^+^ T cells (NCF1, *r* = 0.336, *p* < 0.001; NCF2, *r* = 0.328, *p* < 0.001; and NCF4, *r* = 0.464, *p* < 0.001), B cells (NCF1, *r* = 0.452, *p* < 0.001; NCF2, *r* = 0.425, *p* < 0.001; and NCF4, *r* = 0.265, *p* < 0.001), macrophages (NCF1, *r* = 0.466, *p* < 0.001; NCF2, *r* = 0.672, *p* < 0.001; and NCF4, *r* = 0.559, *p* < 0.001), neutrophils (NCF1, *r* = 0.566, *p* < 0.001; NCF2, *r* = 0.683, *p* < 0.001; and NCF4, *r* = 0.575, *p* < 0.001), and DCs (NCF1, *r* = 0.636, *p* < 0.001; NCF2, *r* = 0.726, *p* < 0.001; and NCF4, *r* = 0.528, *p* < 0.001) (Figures 6(b)–6(d)). Specifically, NCF2 expression was not correlated with immune subtypes (C1-C6: wound healing, IFN-gamma dominant, inflammatory, lymphocyte depleted, and TGF-*β* dominant) in KIRC. In addition, it was found that the mRNA levels of NCF1 and NCF4 were obviously decreased in immunologically quiet KIRC immune subtype.

**Figure 7:**
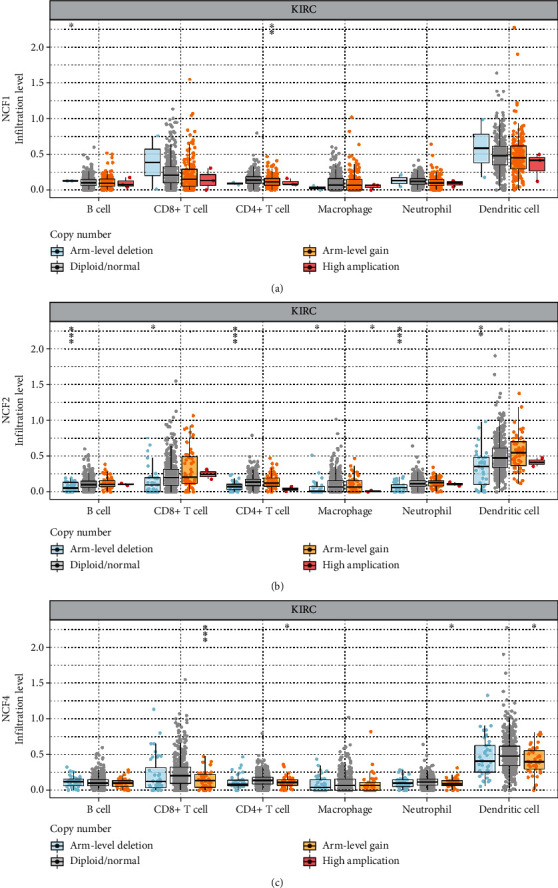
The correlation between copy number alteration of NCFs and immune cell infiltration in KIRC. (a–c) CNA of NCF1, NCF2, and NCF4 had significant correlations with immune infiltration cells including CD4^+^ T cells and B cells. CNA: copy number alteration.

Moreover, the relationships between somatic copy number alterations (SCNA) of the 3 genes and tumor infiltration levels among KIRC were investigated. Interestingly, the results showed that the CNA of NCF1 had significant correlations with the infiltration levels of CD4^+^ T cells and B cells; the CNA of NCF2 had significant correlations with CD8^+^ T cells, CD4^+^ T cells, B cells, neutrophils, dendritic cells, and macrophages; and the CNA of NCF4 had a significant correlation with CD8^+^ T cells, CD4^+^ T cells, neutrophils, and dendritic cells (Figures 7(a)–7(c)).

### 3.7. Enrichment Analyses of NCFs in KIRC

**Figure 8:**
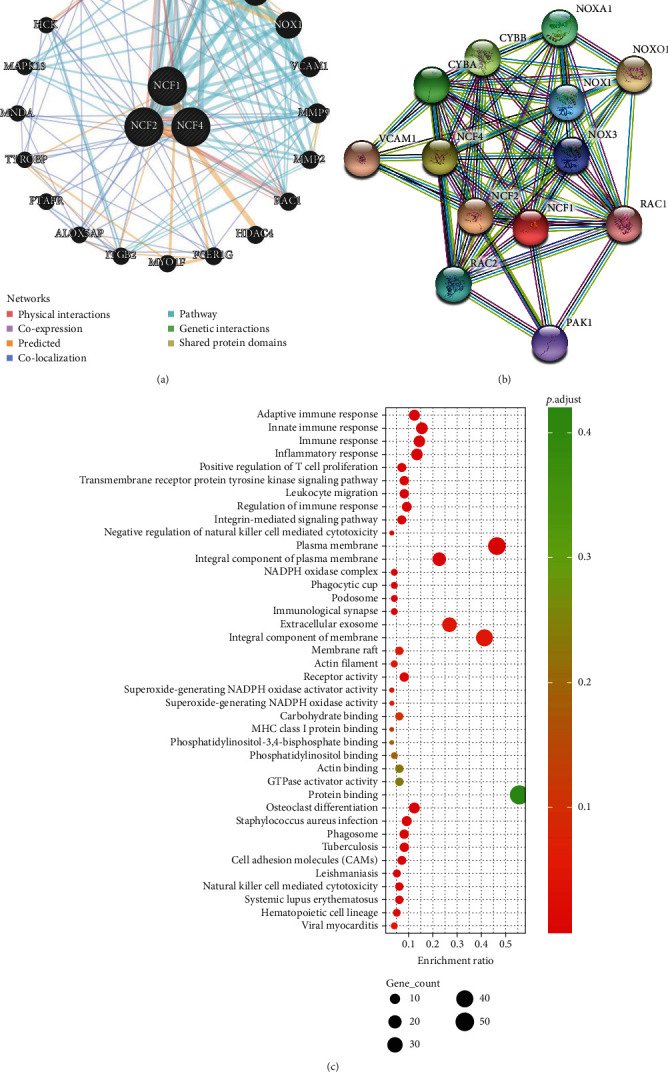
Enrichment pathway of NCFs in KIRC. (a) PPI network of NCF1, NCF2, and NCF4 (GeneMANIA). Different colors of the network edge indicate the bioinformatics methods applied: physical interactions, coexpression, predicted, colocalization, pathway, genetic interactions, and shared protein domains. (b) PPI network of NCF1, NCF2, and NCF4 (STRING). (c) Bubble diagram showed the GO enrichment in cellular component (CC) terms, biological process (BP) terms, and molecular function (MF) terms and bar plot of Kyoto Encyclopedia of Genes and Genomes (KEGG) enriched terms.

We then performed enrichment analyses of NCFs. We collected NCF1, NCF2, and NCF4 and their coexpressed genes and plotted PPI plots with GeneMANIA (20) and STRING (10) (Figures 8(a) and 8(b)). The PPI network was constructed and revealed that NCFs were associated with osteoclast differentiation, natural killer cell-mediated cytotoxicity, MHC class I protein binding, GTPase activator activity, immunological synapse, and immune response.

To understand specifically the potential role of NCFs in the development of kidney renal clear cell carcinoma, we conducted GO and KEGG functional enrichment analysis of the top 100 correlated genes of distinct NCFs using DAVID which was extracted from the GEPIA (http://gepia.cancer-pku.cn/) database as presented in Table S1. We observed that all these three members were closely associated with immune-related biological functions, such as adaptive immune response, innate immune response, regulation of immune response, and integrin-mediated signaling pathway (Figure 8(c)). Therefore, we have reason to believe that NCF members may be related to the immune response in the tumor microenvironment (Figure 8(c)). In addition, we have also observed a higher correlation between NCFs and immunosuppressive immune infiltrating cells (Table 3).

### 3.8. NCF4 Inhibition Blocks Kidney Cancer Cell Proliferation In Vitro

**Figure 9:**
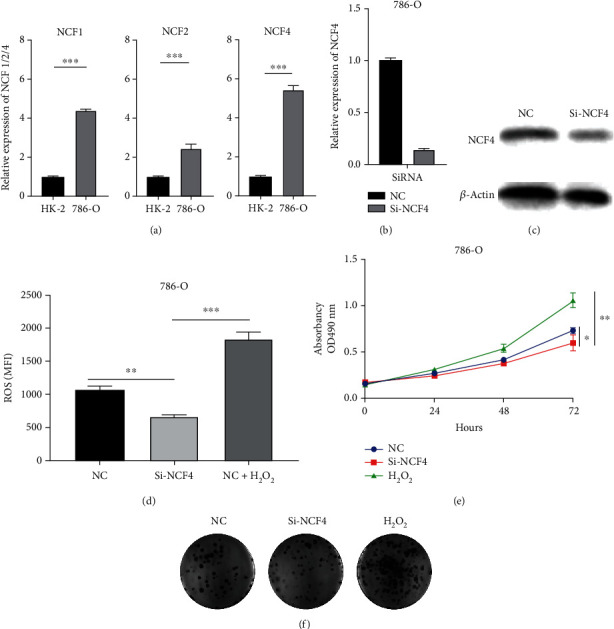
NCF4 promotes KIRC cell proliferation. (a) Expression levels of NCF1, NCF2, and NCF4 in HK-2 and 786-O cell lines were determined by qRT-PCR. NCF4 silencing effect was examined by (b) qRT-PCR and (c) western blotting in 786-O cell lines. After nonlethal oxidative stress induced by 500 *μ*M H_2_O_2_ for 3 hours to 786-O cells and NCF4 knockdown 786-O cells, (d) flow cytometry was used to evaluate the ROS levels, (e) CCK-8 assay was performed to measure cell viability, and colony formation assay was used to test cell proliferation. ^∗∗∗^*p* < 0.001.

We tested the expression of NCFs in human normal renal tubular epithelial cell line HK-2 and KIRC cell line 786-O and found that NCF1, NCF2, and NCF4 were significantly upregulated in the KIRC cell line (Figure 9(a)). To verify the effects of NCF4 on the biological function of KIRC cells in vitro, the siRNAs were synthesized for knocking down the expression of NCF4 in the 786-O cell line (Figures 9(b) and 9(c)). Overexpression of NCF4 led to activation of the NADPH oxidase 2 complex and ROS production (activation of NADPH oxidase subunit NCF4 induces ROS-mediated EMT signaling in kidney cancer cells). The generation, location, and local concentration of free radicals in tumor cells play an important role in the biological behavior of tumors [29]. In order to explore the relationship between them, we measured the level of ROS after knocking down NCF4 in the 786-O cell line. NCF4 knockdown decreases the level of ROS in kidney cancer cells (Figure 9(d)). A CCK-8 assay indicated that cell viability was inhibited by silencing NCF4 and enhanced by exogenous hydrogen peroxide in 786-O cells (Figure 9(e)). The data of the colony formation assay also showed that exogenous hydrogen peroxide increased the proliferation, whereas silencing NCF4 inhibited the proliferation in kidney cancer cells (Figure 9(f)).

### 3.9. NCF4 Inhibition Blocks the Migration and Epithelial-Mesenchymal Transition of Kidney Cancer Cells In Vitro

**Figure 10:**
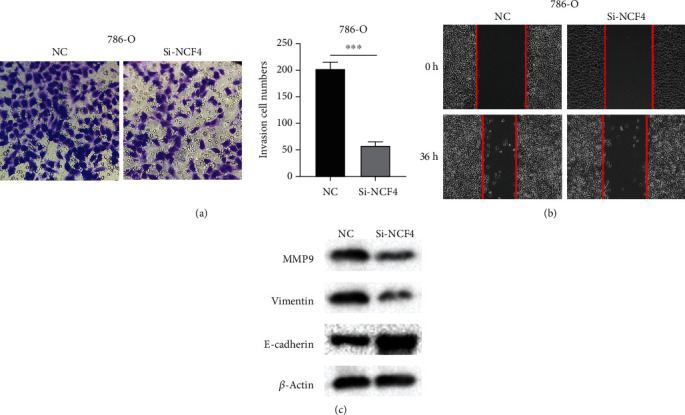
NCF4 promotes KIRC cell epithelial-mesenchymal transition. (a) A transwell assay was applied to detect the effect of NCF4 silencing with respect to the migration of 786-O cells. (b) A wound healing assay was used to detect cell migration of NCF4 knockdown 786-O cells. (c) Effects of NCF4 on the expression levels of EMT-associated proteins in NCF4 knockdown 786-O cells were determined by western blotting. Data represent the mean ± SD of three separate experiments. ^∗∗∗^*p* < 0.001. EMT: epithelial-mesenchymal transition.

Wound healing assays and transwell assays were conducted simultaneously after NCF4 knockdown to figure out the potential role of NCF4 in the migration and invasion capacity of bladder cancer cells. The results showed that knockdown of NCF4 significantly inhibited the ability of migration of KIRC cells (Figures 10(a) and 10(b)). Epithelial-mesenchymal transition (EMT) has been reported to play an important role in carcinoma metastasis, and due to EMT-mediated cell morphology changes, tumor cells are more likely to metastasize to distant places [30]. After confirming that NCF4 mediated KIRC cell migration, we investigated the EMT-relevant markers by western blotting. As the result showed, the expression of the epithelial marker E-cadherin was enhanced, and the mesenchymal markers vimentin and matrix metalloproteinase MMP9 that are closely correlated with metastasis were attenuated when NCF4 was knocked down (Figure 10(c)).

## 4. Discussion

Clear cell RCC (KIRC) is a kind of silent cancer. The symptoms are not obvious in the early stage of the disease and it is difficult to be detected [31]. Therefore, the prognosis of this disease is often very bad [31]. RCC is showing an upward trend all over the world, especially in developed countries that maintain a high incidence [32]. At present, the research on early screening and molecular diagnosis of RCC is continuously deepening, and the diagnosis and treatment of RCC have been improved to a certain extent [33].

RCC is considered to be an immunogenic tumor, but it is known to mediate immune function to a large extent by causing immunosuppressive cells (such as regulatory T cells and suppressor cells of myeloid origin) to infiltrate the tumor microenvironment, considered as a “cold tumor” [34]. With the further in-depth research of TME, the receptors on the surface of immune cells and stromal cells in the tumor microenvironment have been recognized and studied more, which brings more opportunities and approaches for tumor treatment but also clarifies tumor progression. Therefore, more abnormally expressed genes with potential clinical relevance need to be considered for their possible effects, which will bring benefits to patients with more detailed diagnostic evaluation and treatment. In addition, the data of the past few decades clearly show that immunosuppressive changes in tumors are important tumor driving factors and immune escape mechanisms, which occur earlier. Tumor immunosuppression is usually found in cancer. Tumors mainly pass through two types of cells, regulatory T cells or Tregs and myeloid cells, called myeloid-derived suppressor cells (MDSC), which are immunosuppressive agents, which means that they secrete chemicals (such as cytokines) to inhibit infiltrating T cells [35].

In our study, NCF1, NCF2, and NCF4 showed a high expression in renal clear cell carcinoma. And we further proved that especially NCF1 and NCF4 were significantly correlated with high tumor grade and clinic stage. In addition, overexpression of NCF1, NCF2, and NCF4 was associated with shorter overall survival. So far, more and more evidence show that NCF1, NCF2, and NCF4 play an important role in tumorigenesis and development.

NOX2 is often referred to as NADPH oxidase, composed of the assembly of CYBB/gp91phox with the membrane-anchored CYBA/p22phox and the cytosolic subunits NCF4/p40phox, NCF1/p47phox, and NCF2/p67phoxon the plasma membrane to generate extracellular ROS [16]. ROS affects the occurrence, development, and metastasis of cancer through a variety of mechanisms [16]. In recent years, a large number of studies have shown that altered ROS production may promote tumors, while other findings have also proved that ROS production can increase the sensitivity of cancer cells to various death-inducing pathways [36]. NCFs are the subunits of NOX2. Variations and deletions in any one subunit may induce changes in ROS production, which will affect tumors.

The study by van der Weyden et al. showed that NOX2 significantly affects the process of metastasis, because mice that are genetically deprived of any major NOX2 subunits (any subunit is necessary for NOX2 function) always after intravenous injection of tumor cells show a lower rate of lung metastasis [37]. Kelkka et al. found that NCF1 (m1J) mutant mice developed significantly smaller tumors in two melanoma models and tumor incidence was reduced in Lewis lung cancer tumors. The lack of ROS-mediated protection against tumor growth was associated with increasing immunity-associated cytokines [38]. NCF2 is significantly upregulated in RCC, as a potential factor predicting RCC [39].

Immune infiltrating cells in the tumor environment have received more and more attention, and many of their functions have been found to be targets for tumor treatment. As early as 1985, Miller et al. began to study the effect of active immunotherapy for renal cell carcinoma [40]. Gillon et al. explored the role of the immune response reflected in the migration inhibition factor (MIF) test in the defense mechanism of RCC patients and explored the role of macrophages in kidney cancer [41].

The results of expression with NCFs and TIMER database results indicate that these three genes are strongly related to SPI1, HCK, VAV1, CD53, ITGB2, and so on. According to the results of KEGG pathway enrichment, they are mainly involved in inflammation and immune response or tumorigenesis [42–46]. Our results show that NCFs and their coexpressed genes, including SPI1, HCK, VAV1, CD53, and ITGB2, are significantly associated with tumor-infiltrating immune cells, especially with immunosuppressive macrophages. According to the result of the TISIDB database, the mRNA levels of NCF1 and NCF4 were obviously decreased in immunologically quiet KIRC immune subtype. According to the difference in copy number, both arm-level deletion and arm-level gain of NCFs may induce changes in the level of immune cell infiltration. These results indicate that the expression level of NCFs is closely related to tumor-infiltrating immune cells. NCFs and their coexpressed genes may participate in the regulation of the immune response of renal cell carcinoma, leading to a poor prognosis of GC patients.

It is worth mentioning that when exploring the markers related to NCFs and immune cells, we found that NCFs showed a strong correlation with M2 macrophages and tumor-associated macrophages, but not with M1 macrophages. The above data indicate that the high expression level of NCFs may promote the differentiation of macrophages into M2 macrophages and finally into TAM, which may contribute to the occurrence of kidney cancer and lead to a poor prognosis. Overexpression of NCFs may induce the massive production of ROS in the tumor immune microenvironment. Studies have shown that TAM induced by ROS can promote tumor proliferation in vitro [47] and angiogenesis [48]. In addition, the sources of ROS production are also different, partly from the tumor cells themselves, but also from other stromal cells in the microenvironment, such as tumor-associated fibroblasts [49] and neutrophils [50].

However, the comprehensive analysis of the NCF family is carried out using different databases and algorithms, and there are still some limitations. First of all, the prognostic NCF family themselves and their related gene characteristics have not been widely adopted and verified. Considering that there are some potential obstacles to the promotion of sequencing technology in clinical tumor detection, the era when molecular prognostic biomarkers are widely used in clinical practice will take more time. Secondly, the role of the NCF family in tumors is only based on our bioinformatics analysis, and the internal mechanism of its impact on tumors deserves more exploration. Thirdly, as a retrospective study, the main limitation of this study lies in its retrospective nature. Therefore, it is necessary to conduct a multicenter prospective study in the future.

## 5. Conclusion

In summary, we found that the overexpression of NCFs in KIRC is associated with clinical manifestations and predicts a poor prognosis. The high expression of NCFs is closely related to the level of infiltrating immune cells, and it may promote the differentiation of macrophages into TAM. Targeting NCFs, NOX2, and ROS-related pathways may become a new antitumor treatment strategy by regulating immune infiltration. These results bring new insights into NCF-mediated KIRC immune regulation.

## Figures and Tables

**Table 1:** Patient characteristics in different mRNA level of NCFs in KIRC.

Variables	NCF1			NCF2			NCF4		
	High (*n* ≥ 14.49)	Low (*n* ≤ 14.48)	*p* value	High (*n* ≥ 17.07)	Low (*n* ≤ 17.06)	*p* value	High (*n* ≥ 17.09)	Low (*n* ≤ 17.07)	*p* value
*Age*			0.363			0.412			0.243
≤60	140	127		144	123		146	121	
>60	128	136		133	131		131	133	
*Gender*			0.327			0.084			0.023
Male	181	167		191	157		194	154	
Female	87	96		86	97		83	100	
*Race*			0.103			0.039			0.323
White	238	223		244	217		242	219	
Black or African American	22	33		21	34		25	30	
*Grade*			<0.001			<0.001			<0.001
1+2	96	147		108	135		103	140	
3+4	171	109		168	112		172	108	
*Pathologic_T*			0.026			0.044			0.053
1+2	154	187		161	180		164	177	
3+4	105	85		107	83		108	82	
*Pathologic_N*			0.004			0.138			0.031
0	121	119		134	105		128	111	
1	14	2		12	4		13	3	
*Pathologic_M*			0.006			0.187			0.036
0	206	216		222	200		210	212	
1	10	1		8	3		9	2	
*Stage*			0.075			0.157			0.051
i+ii	137	160		150	147		139	158	
iii+iv	69	55		72	52		71	53	

**Table 2:** Enrichment of NCF1, NCF2, and NCF4 in KIRC (DAVID). The diagram showed the GO enrichment in cellular component (CC) terms, biological process (BP) terms, and molecular function (MF) terms and bar plot of Kyoto Encyclopedia of Genes and Genomes (KEGG) enriched terms.

Gene	Category	Gene set	Description	Size	ES	*p* value	FDR
NCF1	BP	GO:0002250	Adaptive immune response	121	0.75491	<0.001	<0.001
		GO:0002449	Lymphocyte-mediated immunity	85	0.73747	<0.001	<0.001
		GO:0002694	Regulation of leukocyte activation	167	0.70281	<0.001	<0.001
		GO:0007159	Leukocyte cell-cell adhesion	112	0.72889	<0.001	<0.001
		GO:0002764	Immune response-regulating signaling pathway	159	0.70894	<0.001	<0.001
	CC	GO:0001772	Immunological synapse	32	0.89059	<0.001	<0.001
		GO:0042611	MHC protein complex	19	0.92416	<0.001	0.0016732
		GO:0098636	Protein complex involved in cell adhesion	35	0.83931	<0.001	0.0037646
		GO:0070820	Tertiary granule	155	0.8063	<0.001	0.0046849
		GO:0001891	Phagocytic cup	21	0.8995	<0.001	0.0050195
	MF	GO:0042287	MHC protein binding	24	0.923	<0.001	<0.001
		GO:0003823	Antigen binding	52	0.84395	<0.001	0.0033726
		GO:0019865	Immunoglobulin binding	22	0.91359	<0.001	0.0036792
		GO:0035586	Purinergic receptor activity	26	0.86795	0.0023095	0.0040471
		GO:0052813	Phosphatidylinositol bisphosphate kinase activity	74	0.82566	<0.001	0.0044676
	KEGG	hsa05150	*Staphylococcus aureus* infection	52	0.92179	<0.001	<0.001
		hsa05416	Viral myocarditis	56	0.90004	<0.001	<0.001
		hsa05320	Autoimmune thyroid disease	50	0.90708	<0.001	<0.001
		hsa05140	Leishmaniasis	71	0.89	<0.001	<0.001
		hsa04940	Type I diabetes mellitus	41	0.90434	<0.001	<0.001
NCF2	BP	GO:0009595	Detection of biotic stimulus	21	0.95106	<0.001	<0.001
		GO:0033108	Mitochondrial respiratory chain complex assembly	68	-0.66273	<0.001	<0.001
		GO:0010257	NADH dehydrogenase complex assembly	49	-0.73433	<0.001	<0.001
		GO:0042116	Macrophage activation	78	0.8476	<0.001	0.00047859
		GO:0042107	Cytokine metabolic process	106	0.848	<0.001	0.0004994
	CC	GO:0042611	MHC protein complex	19	0.93646	<0.001	<0.001
		GO:0030964	NADH dehydrogenase complex	43	-0.65251	<0.001	<0.001
		GO:0098798	Mitochondrial protein complex	213	-0.5402	<0.001	<0.001
		GO:0001772	Immunological synapse	32	0.87477	<0.001	0.00058165
		GO:0070820	Tertiary granule	155	0.84456	<0.001	0.00087247
	MF	GO:0038187	Pattern recognition receptor activity	20	0.94123	<0.001	<0.001
		GO:0042287	MHC protein binding	24	0.91201	<0.001	0.00094517
		GO:0003823	Antigen binding	52	0.87062	<0.001	0.00094517
		GO:0019865	Immunoglobulin binding	22	0.93547	<0.001	0.0012602
		GO:0043394	Proteoglycan binding	33	0.87104	<0.001	0.0040957
	KEGG	hsa05150	*Staphylococcus aureus* infection	52	0.92654	<0.001	<0.001
		hsa05140	Leishmaniasis	71	0.90715	<0.001	<0.001
		hsa05310	Asthma	28	0.93861	<0.001	<0.001
		hsa05322	Systemic lupus erythematosus	122	0.86618	<0.001	<0.001
		hsa04640	Hematopoietic cell lineage	93	0.89299	<0.001	<0.001
NCF4	BP	GO:0002250	Adaptive immune response	368	0.78931	<0.001	<0.001
		GO:0032609	Interferon-gamma production	102	0.79916	<0.001	<0.001
		GO:0032613	Interleukin-10 production	46	0.82482	<0.001	<0.001
		GO:0042110	T cell activation	439	0.76892	<0.001	<0.001
		GO:0007159	Leukocyte cell-cell adhesion	310	0.76451	<0.001	<0.001
	CC	GO:0001772	Immunological synapse	32	0.83629	<0.001	<0.001
		GO:0042579	Microbody	125	-0.56583	<0.001	<0.001
		GO:0001891	Phagocytic cup	21	0.85403	<0.001	0.00053463
		GO:0070820	Tertiary granule	155	0.72836	<0.001	0.00080194
		GO:0042611	MHC protein complex	19	0.84613	<0.001	0.0016039
	MF	GO:0019865	Immunoglobulin binding	22	0.88392	<0.001	<0.001
		GO:0016903	Oxidoreductase activity, acting on the aldehyde or oxo group of donors	43	-0.62542	<0.001	<0.001
		GO:0042287	MHC protein binding	24	0.85887	<0.001	0.0011573
		GO:0004896	Cytokine receptor activity	88	0.75566	<0.001	0.001736
		GO:0019955	Cytokine binding	119	0.69907	<0.001	0.009982
	KEGG	hsa05150	*Staphylococcus aureus* infection	52	0.88097	<0.001	<0.001
		hsa04640	Hematopoietic cell lineage	93	0.80875	<0.001	<0.001
		hsa05320	Autoimmune thyroid disease	50	0.84733	<0.001	<0.001
		hsa04672	Intestinal immune network for IgA production	45	0.83546	<0.001	<0.001
		hsa05140	Leishmaniasis	71	0.80899	<0.001	<0.001

**Table 3:** Correlation analysis between NCF1, NCF2, and NCF4 and gene markers of immune cells.

Description	Gene markers	NCF1				NCF2				NCF4			
		None		Purity		None		Purity		None		Purity	
		Cor	*p*	Cor	*p*	Cor	*p*	Cor	*p*	Cor	*p*	Cor	*p*
CD8 T cell	CD8A	0.612	∗∗∗	0.574	∗∗∗	0.436	∗∗∗	0.393	∗∗∗	0.482	∗∗∗	0.403	∗∗∗
	CD8B	0.606	∗∗∗	0.572	∗∗∗	0.405	∗∗∗	0.362	∗∗∗	0.488	∗∗∗	0.424	∗∗∗
T cell (general)	CD3D	0.675	∗∗∗	0.642	∗∗∗	0.446	∗∗∗	0.399	∗∗∗	0.601	∗∗∗	0.536	∗∗∗
	CD3E	0.694	∗∗∗	0.661	∗∗∗	0.462	∗∗∗	0.413	∗∗∗	0.592	∗∗∗	0.527	∗∗∗
	CD2	0.702	∗∗∗	0.672	∗∗∗	0.510	∗∗∗	0.471	∗∗∗	0.600	∗∗∗	0.538	∗∗∗
B cell	CD19	0.448	∗∗∗	0.404	∗∗∗	0.282	∗∗∗	0.247	∗∗∗	0.536	∗∗∗	0.492	∗∗∗
	CD79A	0.503	∗∗∗	0.455	∗∗∗	0.316	∗∗∗	0.265	∗∗∗	0.538	∗∗∗	0.482	∗∗∗
Monocyte	CD86	0.735	∗∗∗	0.715	∗∗∗	0.825	∗∗∗	0.826	∗∗∗	0.704	∗∗∗	0.679	∗∗∗
	CD115 (CSF1R)	0.684	∗∗∗	0.665	∗∗∗	0.685	∗∗∗	0.677	∗∗∗	0.738	∗∗∗	0.720	∗∗∗
TAM	CCL2	0.129	∗∗	0.087	0.062	0.182	∗∗∗	0.154	∗∗	0.130	∗∗	0.077	0.100
	CD68	0.545	∗∗∗	0.528	∗∗∗	0.613	∗∗∗	0.618	∗∗∗	0.484	∗∗∗	0.493	∗∗∗
	IL10	0.527	∗∗∗	0.469	∗∗∗	0.639	∗∗∗	0.603	∗∗∗	0.600	∗∗∗	0.558	∗∗∗
M1 macrophage	INOS (ISYNA1)	-0.070	0.105	-0.113	∗	-0.198	∗∗∗	-0.238	∗∗∗	0.068	0.116	0.023	0.622
	IRF5	0.524	∗∗∗	0.522	∗∗∗	0.442	∗∗∗	0.463	∗∗∗	0.406	∗∗∗	0.411	∗∗∗
	cox2 (PTGS2)	-0.015	0.733	-0.057	0.219	0.108	∗	0.089	0.056	0.163	∗∗∗	0.117	∗
M2 macrophage	CD163	0.483	∗∗∗	0.458	∗∗∗	0.671	∗∗∗	0.664	∗∗∗	0.567	∗∗∗	0.551	∗∗∗
	VSIG4	0.597	∗∗∗	0.578	∗∗∗	0.636	∗∗∗	0.624	∗∗∗	0.703	∗∗∗	0.686	∗∗∗
	MS4A4A	0.530	∗∗∗	0.496	∗∗∗	0.698	∗∗∗	0.693	∗∗∗	0.646	∗∗∗	0.619	∗∗∗
Neutrophils	CD66b (CEACAM8)	0.039	0.374	0.045	0.335	0.113	∗∗	0.136	∗∗	0.074	0.087	0.105	∗
	CD11b (ITGAM)	0.711	∗∗∗	0.690	∗∗∗	0.780	∗∗∗	0.767	∗∗∗	0.657	∗∗∗	0.637	∗∗∗
	CCR7	0.540	∗∗∗	0.496	∗∗∗	0.463	∗∗∗	0.429	∗∗∗	0.579	∗∗∗	0.540	∗∗∗
Natural killer cell	KIR2DL1	0.125	∗∗	0.108	∗	0.102	∗	0.053	0.260	0.096	∗	0.033	0.481
	KIR2DL3	0.134	∗∗	0.152	∗∗	0.131	∗∗	0.118	∗	0.126	∗∗	0.110	∗
	KIR2DL4	0.273	∗∗∗	0.260	∗∗∗	0.169	∗∗∗	0.123	∗∗	0.282	∗∗∗	0.239	∗∗∗
	KIR2DS4	0.075	0.082	0.063	0.175	0.038	0.386	-0.005	0.914	0.093	∗	0.072	0.120
	KIR3DL1	0.121	∗∗	0.144	∗∗	0.131	∗∗	0.116	∗	0.088	∗	0.075	0.107
	KIR3DL2	0.184	∗∗∗	0.174	∗∗∗	0.110	∗	0.091	0.051	0.165	∗∗∗	0.146	∗∗
	KIR3DL3	0.130	∗∗	0.098	∗	0.073	0.092	0.056	0.231	0.144	∗∗	0.122	∗∗
Dendritic cell	HLA-DPA1	0.702	∗∗∗	0.672	∗∗∗	0.697	∗∗∗	0.680	∗∗∗	0.576	∗∗∗	0.522	∗∗∗
	HLA-DPB1	0.763	∗∗∗	0.749	∗∗∗	0.686	∗∗∗	0.667	∗∗∗	0.640	∗∗∗	0.609	∗∗∗
	HLA-DQB1	0.487	∗∗∗	0.442	∗∗∗	0.419	∗∗∗	0.376	∗∗∗	0.402	∗∗∗	0.341	∗∗∗
	HLA-DRA	0.731	∗∗∗	0.714	∗∗∗	0.750	∗∗∗	0.741	∗∗∗	0.612	∗∗∗	0.583	∗∗∗
	BDCA-1 (CD1C)	0.395	∗∗∗	0.345	∗∗∗	0.460	∗∗∗	0.419	∗∗∗	0.429	∗∗∗	0.379	∗∗∗
	BDCA-4 (NRP1)	-0.001	0.982	-0.064	0.167	0.212	∗∗∗	0.166	∗∗∗	0.050	0.247	-0.015	0.756
	CD11c (ITGAX)	0.655	∗∗∗	0.629	∗∗∗	0.676	∗∗∗	0.670	∗∗∗	0.645	∗∗∗	0.639	∗∗∗
Th1	T-bet (TBX21)	0.455	∗∗∗	0.434	∗∗∗	0.297	∗∗∗	0.261	∗∗∗	0.426	∗∗∗	0.383	∗∗∗
	STAT4	0.454	∗∗∗	0.404	∗∗∗	0.373	∗∗∗	0.342	∗∗∗	0.523	∗∗∗	0.465	∗∗∗
	IFN-*γ* (IFNG)	0.592	∗∗∗	0.553	∗∗∗	0.410	∗∗∗	0.365	∗∗∗	0.453	∗∗∗	0.382	∗∗∗
	TNF-*α* (TNF)	0.478	∗∗∗	0.475	∗∗∗	0.388	∗∗∗	0.384	∗∗∗	0.462	∗∗∗	0.437	∗∗∗
	STAT1	0.588	∗∗∗	0.537	∗∗∗	0.633	∗∗∗	0.605	∗∗∗	0.428	∗∗∗	0.352	∗∗∗
Th2	GATA3	0.212	∗∗∗	0.234	∗∗∗	0.133	∗∗	0.118	∗	0.222	∗∗∗	0.188	∗∗∗
	STAT6	0.150	∗∗∗	0.167	∗∗∗	0.189	∗∗∗	0.206	∗∗∗	0.099	∗	0.138	∗∗
	STAT5A	0.629	∗∗∗	0.614	∗∗∗	0.598	∗∗∗	0.583	∗∗∗	0.535	∗∗∗	0.495	∗∗∗
	IL13	0.053	0.219	0.038	0.420	-0.063	0.145	-0.061	0.194	0.173	∗∗∗	0.165	∗∗∗
Tfh	BCL6	-0.029	0.500	-0.034	0.465	0.029	0.499	0.028	0.544	0.091	∗	0.098	∗

## Data Availability

The datasets analyzed in the current study are publicly available in cBioPortal and TCGA and accessed via the Genomic Data Commons Data Portal (https://gdc.cancer.gov). The original contributions presented in the study are included in the article and within the Supplementary Materials.

## Supplementary Materials

Supplementary Materials Table S1: 100 top coexpression genes with NCFs. NCFs' coexpressed genes (100) were identified from GEPIA2 and conducted pathway enrichment analyses via the DAVID tool.
