# Supplementary material for: NCF1/2/4 Are Prognostic Biomarkers Related to the Immune Infiltration of Kidney Renal Clear Cell Carcinoma
Source: Biomed Res Int. 2021 Oct 18;2021:5954036. doi: 10.1155/2021/5954036 (PMC8545530; doi:10.1155/2021/5954036)
Supplement: Supplementary Materials — Table S1: 100 top coexpression genes with NCFs. NCFs' coexpressed genes (100) were identified from GEPIA2 and conducted pathway enrichment analyses via the DAVID tool. [file 5954036.f1.docx]

Table S1 100 top co-expression genes with NCFs

| SPI1 | WAS | ARHGAP30 | AIF1 | C1QC | RGS19 | OSCAR | NLRC4 | FAM78A | APOBR |
| --- | --- | --- | --- | --- | --- | --- | --- | --- | --- |
| HCK | SASH3 | CD300LF | LILRB2 | SH3BP1 | SNX20 | PARVG | HLA-DRA | LILRA6 | MILR1 |
| VAV1 | SELPLG | DOCK2 | CD37 | ARRB2 | GPSM3 | C1QB | WDFY4 | CD48 | RPL7AP64 |
| CD53 | LAT2 | CD33 | AC011899.9 | TNFAIP8L2 | TBXAS1 | SLC15A3 | LY86 | SLAMF8 | CLEC7A |
| PILRA | CD4 | CYTH4 | SIGLEC9 | PLEK | HLA-DMB | ARL11 | BTK | RASGRP4 | SIGLEC1 |
| ITGB2 | FCER1G | MS4A6A | MYO1F | HCLS1 | LAIR1 | CTSS | IKZF1 | LGALS9 | LILRB1 |
| LAPTM5 | AMICA1 | NFAM1 | LPXN | CD86 | SCIMP | CORO1A | AD000671.6 | CD84 | MNDA |
| NCF4 | NCKAP1L | TYROBP | NCF1B | FMNL1 | BIN2 | EVI2B | C1QA | GPR141 | RNASE6 |
| NCF1C | DOK2 | LILRB4 | FERMT3 | DOK3 | AOAH | IGSF6 | RP5-1091N2.9 | SAMHD1 | FPR3 |
| NCF2 | IL10RA | LCP1 | GMIP | CLEC4A | STAC3 | C3AR1 | LCP2 | CSF1R | GPR65 |

NCFs’ co-expressed genes (100) were identified from GEPIA2 and conducted pathway enrichment analyses via the DAVID tool.
